# Research on military-civilian collaborative innovation of science and technology based on a stochastic differential game model

**DOI:** 10.1371/journal.pone.0292635

**Published:** 2024-01-05

**Authors:** Xin Liang, Yunjuan Liang, Weijia Kang, Hua Wei

**Affiliations:** Department of Management Engineering and Equipment Economics, Naval University of Engineering, Wuhan, China; University of Madeira / NOVA Lincs, PORTUGAL

## Abstract

The construction of an integrated national strategic system and capability is an essential goal of implementing the strategy of military-civilian integration in the contemporary era. And the collaborative innovation of military-civilian S&T is an inevitable choice to achieve this goal. Due to the dynamic, complex, and stochastic characteristics of military-civilian S&T collaborative innovation, the level of S&T innovation is highly volatile. This paper takes the internal and external stochastic disturbance factors of military-civilian S&T collaborative innovation as the perspective, studies the strategy selection problem of military-civilian S&T collaborative innovation under military domination, constructs a differential game model to explore the innovation strategies under the non-cooperative model without military subsidies, the non-cooperative model with military subsidies, and the collaborative model. Finally, we use numerical experiments to verify the validity of the conclusions. The study shows that: (1) Within a reasonable range of values of the benefit distribution coefficient, the system can achieve the Pareto optimum, and the collaborative model is conducive to improving the S&T innovation level and the optimum benefit level of the system. (2) Military subsidies can increase the benefits of the system and the parties involved to achieve Pareto improvement. (3) The level of S&T innovation under the collaborative model has dynamic evolutionary characteristics of maximum expectation and variance. As the intensity of disturbance increases, the stability of the system may be destroyed. Risk-averse civil enterprises prefer the cooperative mode, whereas risk-averse civil enterprises prefer the non-cooperative model.

## 1 Introduction

Building an integrated national strategic system and capability means realizing the integrated planning, overall promotion, and integrated application of economic construction and national defense construction so as to maximize the overall national strategic benefits. The most critical is to combine the innovation-driven development strategy with the strategy of strengthening the military through science and technology, so as to consolidate and improve the integrated military-civilian S&T innovation system and independent innovation capability. Military-civilian S&T collaborative innovation is precisely the intersection of two strategies: innovation-driven and S&T-enhanced military. As an important component of the integrated national strategic system and capability, S&T collaborative innovation plays an important leading and supporting role in improving the integrated national strategic system and capability.

At present, military-civilian S&T collaborative innovation has become the core military technology innovation strategy of China. Facing the current transformation requirements of intelligent warfare [1], artificial intelligence (AI) has become an emerging dual-use technology [2], and military applications of AI such as cloud computing, big data analysis, quantum computing, and autonomous systems urgently need to strengthen military-civilian S&T collaborative innovation [3, 4]. For example, quantum innovation technology, which has received considerable attention in the field of frontier S&T innovation, lags behind the civilian sector in its defense applications [5, 6]. In 2017, the *CAS innovative center for quantum information and quantum physics* (Shanghai) jointly released a quantum computing cloud platform with Alibaba Cloud Computing. Alibaba, as a civilian company, participated in the cutting-edge strategy of China’s defense innovation system for the first time, marking the beginning of a new trend of civilian companies participating in cutting-edge innovation [7]. The *Tianhe* supercomputer is another typical example of successful military-civilian S&T collaborative innovation. Based on China’s Advanced Technology Research and Development Program (863 Program), the *Tianhe* series supercomputers were mainly developed by the *National University of Defense Technology* (military) in the early stage, and introduced to the *National Supercomputing Center* through military-civilian coordination to build a technology application platform in the application stage, with enterprise groups such as *China National Petroleum Corporation* (CNPC), *Sinopec*, and *BGI* (Shenzhen) as end users. The technology was improved and upgraded by the *National University of Defense Technology* based on feedback from applications. This process has promoted both the transformation and application of defense science and technology achievements in the civilian market, as well as the in-depth development of defense technology innovation, and is a model for the implementation of military-civilian S&T collaborative innovation in China.

Thus, it can be seen that China’s military-civilian S&T collaborative innovation has achieved remarkable results. However, due to the relative specificity, complexity and uncertainty of military needs, it is difficult to form traction between military and civilian needs, resulting in the lack of coordination and innovation in the military and civilian fields. Low investment of civilian enterprises in the field of scientific research has also limited the improvement of independent innovation capability and competitiveness. Therefore, the lack of military-civilian collaborative innovation capability is a highly prominent obstacle to the construction and development of China’s military.

In the integrated context of S&T collaborative innovation system, the long-term, stochastic and complex nature of S&T innovation makes the innovation collaboration between military and civilian enterprises show a dynamic trend. In order to maintain sustainable core competitiveness, the military and civilian sides need to conduct continuous S&T collaborative innovation, so the continuous time variable is an influential factor in the decision making of S&T innovation behavior. General game theory is unable to solve dynamically evolving problems. As an important dynamic model for dealing with conflict, competition, or cooperation between multiple parties in continuous time [8], the differential game is suitable for studying the optimal decision behavior under the direct interaction of the behavior of multiple participants in the system [9]. Therefore, this paper applies a stochastic differential game model based on different scenarios of military-civilian S&T collaborative innovation with the military (military enterprises) and civilian enterprises as the main participants from the perspective of the temporal continuity and stochastic nature of S&T collaborative innovation, and considers the influence of military subsidies on the incentive effect of innovation. The purpose of this paper is to establish a reasonable and practical theoretical model to study the problem of military-civilian S&T collaborative innovation in the context of integrated national strategic system and capability, and to provide an effective reference for improving the policy system of military-civilian S&T collaborative innovation.

The remainder of this paper is organized as follows. Section 2 reviews the significant literature in the related field. Section 3 develops a differential game model of military-civilian S&T collaborative innovation. Section 4 presents a comparative analysis of the equilibrium results under three different scenarios. Section 5 performs numerical simulation experiments to verify the plausibility of the model. Section 6 provides the main conclusions and policy recommendations.

## 2 Literature review

The report of the 19th National Congress of the Communist Party of China clearly puts forward the goal of forming a pattern of in-depth development of military-civilian integration and building an integrated national strategic system and capability. Constructing an integrated national strategic system and capability is the ultimate aim to be accomplished after forming an *all-factor*, *multi-discipline and high-efficiency pattern of deep military-civilian integration development* [10, 11]. Its essence is to promote the economic development and national defense construction from the decentralized design to the military-civilian integration, from the key areas to the extension and expansion of multiple fields, as well as from the loose combination of elements to the integration of all elements, thereby realizing the process of structural optimization, resource conservation and technological innovation [12]. Modern warfare has put forward more demands for military science and technology innovation. China faces the double pressure of catching up with frontier technology and attacking key core technology. However, the industrial structure and factor structure can hardly adapt to the demand upgrade, and the self-enclosed development model can no longer meet the requirements of military competition and S&T innovation. In the background of globalization, commercialization and digital economy, S&T innovation presents significant features such as cross-boundary integration and collaborative association, and the development of military-civilian S&T collaborative innovation has become an inevitable trend [13, 14].

With the development of economy and society, the development of integration between industries or organizations has become a common phenomenon, the essence of which is to break through the boundaries and barriers in terms of demand, technology, products and skills to achieve resource sharing [14]. Collaboration between system entities is also more likely to result in innovation [15]. This type of collaborative innovation is characterized by a wide range of interactive and integrated innovation activities among enterprises, the public sector, universities and research institutions, and non-profit organizations to achieve scientific and technological innovation [16–18]. Existing studies show that collaborative innovation is considered an important tool for solving current major social problems [19], especially in the public domain [20]. The overall positive effect of external knowledge on enterprises’ innovation activities has also been well documented [21–24]. Collaborative innovation promotes knowledge complementarity, resulting in faster and higher quality innovation as well as higher productivity [25, 26]. In addition, collaborative innovation relationships formed between subjects through resource sharing, information exchange, and cooperative interaction can achieve improved innovation performance [17]. As a consequence, collaborative innovation has become a vital innovation strategy [27] and an effective approach for conducting S&T innovation [13, 25, 28]. For S&T innovation in the military sector, the generation of new military S&T achievements requires multiple stages such as basic research, S&T research, and innovation industry chain integration, etc. With the increasing complexity of weapon and equipment systems, many technological innovations cannot rely on the military to complete independently, and the entry of civilian enterprises into the military market (CMEE-MPM) can effectively improve defense S&T capability and efficiency [29]. Indeed, collaborative innovation is an effective way to address this problem.

The problems related to military-civilian S&T collaborative innovation have traditionally attracted great attention. The existing literature has explored the influencing factors, economic effects and mechanisms of military-civilian S&T collaborative innovation from macro and micro dimensions, respectively.

In terms of theory and policy, foreign research on military-civilian S&T collaborative innovation mainly focuses on standard establishment, system construction, system design, and so on. Brandt [30] proposed the concept of dual-use technology and its application in the defense domain. Lazaric et al. [31] argued that the defense innovation system mainly consists of two major groups of interacting military and civilian participants, and technology and system change and creativity enhancement are the urgent problems to be addressed. Acosta et al. [32] concluded that policy research on dual-use technologies focuses on linking military and civilian technologies and promoting S&T innovation with military and civilian components.

Domestic related literature mainly evaluated the level of military-civilian collaboration development, military-civilian collaboration policies and technology integration models in China. The overall level of military-civilian integration development in China has entered the middle level, but there are some differences in the development level of various areas [33]. Wang et al. [34] found that there is a large gap between different levels of military-civilian integration policies through quantitative evaluation of military-civilian integration policies. Cao et al. [35] found that the barriers of military-civilian technology integration affect the efficiency of technology integration.

At the industrial level, the existing literature has mainly studied the factors influencing the technological collaborative innovation capability of the military and civilian industries [36]. Mowery [37] concluded that relying on external contractors would improve the performance of military-civilian S&T innovation and promote wider dissemination and application of innovation fruits. Kulve and Smit [38] proposed to construct a dual capability network and make it an important part of achieving the strategic development of military-civilian S&T integration. Zhou [39] conducted a study on the influence of technological collaborative innovation capability of military-civilian integration industries. Tian et al. [40] constructed a system dynamics model by analyzing the interaction between influencing factors based on the establishment of a system of influencing factors for collaborative innovation in military-civilian S&T. Lu et al. [41] applied a network slack-based metric (NSBM) DEA model to evaluate the R&D efficiency and socio-economic efficiency of dual-use technology innovation programs, Lee and Park [42] further built on it to analyze the efficiency of the whole weapon system in terms of technical capabilities and operations.

At the empirical analysis level, the existing literature addresses the performance of military-civilian S&T collaborative innovation. Yang et al. [43] employed panel data from Chinese provinces to empirically analyze the impact of military-civilian collaborative innovation on defense innovation performance and spatial spillovers. Stanley-Lockman [44] assessed the differences in the pursuit of open military-civilian innovation approaches and systems among different military services in the U.S. Sun et al. [45] used a double-difference method to examine the impact and path of military-civilian collaboration on firms’ key core technology innovation, and provided empirical studies on the impact of military-civilian collaboration strategy on the breakthrough of key core technologies.

From the perspective of policy practice, countries worldwide to actively promote the implementation of new initiatives to encourage collaborative innovation in military-civilian S&T. The U.S. Department of Defense (DoD) has established the DoD Office of Technology Transfer (OTT) to implement the Defense Technology Transfer Program (DTTP), which is responsible for developing technology transfer and dual-use technology policies. In particular, the Defense Advanced Research Projects Agency (DARPA) has designed good collaboration mechanisms and established an innovation ecosystem including academic organizations, S&T companies, and government partners that have played a major role in driving the U.S. military-civilian integration (MCI) process. Russia has actively established joint military-civilian groups and emphasized the development and utilization of dual-use technologies. Japan has mainly formulated policies and regulations to ensure the development of military-civilian integration, and promoted the integration of industry-military-academia. Israel has established a bidirectional military-civilian integration ecosystem model, which effectively promotes the interaction and integration of defense S&T and civilian industrial technology. The advanced practices in the implementation of military-civilian integration policies in the above-mentioned countries have significant implications for the construction of China’s military-civilian S&T innovation system.

Summarizing the above literature, we found that the current degree of integration of the military-civilian S&T innovation system in China is still not deep enough. The highlighted problems, such as the poor operability of policy measures, the complicated innovation procedures of military technologies, and the ineffective information sharing between the military and civilian sectors, have to some extent hindered the momentum of collaborative military-civilian S&T innovation.

The differential game has been widely used in the study of collaborative innovation. Guo et al. [46] presented a method to calculate the optimal effort level and optimal revenue of both parties in the school-enterprise collaborative innovation (SECI) system based on the differential game, which effectively argued the advantages of school-enterprise collaborative innovation in DT technology. Xu and Fan [47] applied the differential game theory to explore the problems of technology innovation and social responsibility in school-enterprise collaboration. Yi and Zhang [48] employed the differential game approach to examine the utility of multi-channel financing on the benefits of green technology innovation in industry-university research under different cooperation models and its impact on the distribution of benefits. Cheng et al. [49] developed a green supply chain differential game model for green technology R&D and compared the equilibrium solutions under centralized and decentralized decision making. Yin and Li [50] studied the stochastic differential game problem of green building technology transfer from academic research institutes to construction firms in a construction firm-academic research institute collaborative innovation (BACI) system. Wang et al. [51] considered the reverse supply chain differential game problem of technological innovation from a competitive perspective. Ma et al. [52] studied the optimal knowledge sharing strategy among firms in collaborative innovation in industrial clusters based on the differential game, and found that the Pareto optimum of the individual returns of both parties can be achieved within a certain threshold of the revenue allocation coefficient. Wen et al. [53] built a dynamic differential game model for innovation strategies and behavioral choices in competitive pharmaceutical supply chains. The application of differential game in the field of military-civilian collaborative innovation is comparatively less. Cao et al. [54] applied stochastic differential game theory to build a dynamic development model of dual-use technology conversion. Zhao et al. [55] constructed a differential game model to study technology sharing between military and civilian enterprises in the military-civilian collaborative innovation system, and found that collaborative cooperation is strictly better than the non-cooperative scenario.

There are some limitations in the existing literature. (1) Most of the existing studies focus on discussing the theoretical framework, development model and countermeasure mechanism of military-civilian S&T collaborative innovation from a macroscopic perspective, but this paper will introduce the differential game approach to study the problem of practical strategy selection in military-civilian S&T collaborative innovation. (2) Unlike the research on school-enterprise collaborative innovation and industry-university-research collaborative innovation, the military often occupies a dominant position in China’s military-civilian collaborative innovation system, and the literature on military-civilian S&T innovation based on the differential game model only considers military enterprises and local enterprises, and only introduces military behavior as an exogenous variable in the game model. Therefore, this paper introduces military enterprises as military affiliates and military cost subsidies as endogenous variables into the model for analysis. (3) The existing literature research does not fully reflect the important characteristics of uncertainty and randomness of technological innovation, which makes the research conclusions have certain limitations. This paper focuses on the influence of random disturbance variables and decision makers’ risk preferences on the choice of military-civilian S&T collaborative innovation strategy.

In summary, the main problem that military-civilian S&T collaborative innovation aims to solve is how to stimulate both the military and civilian sides to maximize the innovation level and benefits of the collaborative innovation system. Since S&T innovation is usually long-term, complex and high-risk, especially the barriers for civilian high-tech enterprises to participate in collaborative innovation, the rapid change of technological products and the uncertainty of the external environment often make the risks of military-civilian S&T collaborative innovation unpredictable. In the process of military-civilian S&T collaborative innovation, military and civilian enterprises have conflicting goals, both aiming to maximize their own interests. In order to motivate civilian enterprises to participate in innovation, the military makes up for civilian enterprises’ innovation cost expenditures to a certain extent, but the cost subsidies are also limited by military budget funds. Accordingly, this paper introduces the differential game approach from the perspective of the long-term and stochastic nature of military-civilian S&T collaborative innovation, and investigates the strategy selection in military-civilian S&T collaborative innovation from a dynamic perspective, using the HJB equation to examine the optimal innovation effort level, optimal innovation benefit level, and optimal total innovation benefit level of military and civilian enterprises under three military-civilian S&T innovation modes. The paper further explores the key factors affecting the choice of military-civilian S&T collaborative innovation models, analyzes the effect of military cost subsidies on military-civilian S&T collaborative innovation, investigates the effects of random disturbance variables and decision makers’ risk preferences on strategies, and seeks the optimal strategies for military-civilian S&T collaborative innovation under a dynamic framework.

The main innovations of this paper are as follows. (1) The existing literature research fails to fully reflect the important characteristics of uncertainty and randomness of technological innovation, which makes the research conclusions have certain limitations. This paper introduces stochastic factors into the military-civilian science and technology innovation model for research, and analyzes the influence of uncertainty on strategy selection. (2) The improvement effect of military subsidies on the decision making and overall benefits of military-civilian S&T collaborative innovation is analyzed. (3) The impact of stochastic disturbances of different intensity on system stability is revealed. It is expected that the obtained relevant conclusions can provide support for rational theoretical basis and policy reference for scientific decision making in strategy selection, incentive mechanism and benefit distribution of military-civilian S&T collaborative innovation, and promote the development of military-civilian S&T collaborative innovation.

## 3 Model construction

As an important game method to study how participants interact and make decisions in continuous time, a differential game can reflect the dynamic change of the decision-making subject’s strategy. In order to fully reflect the important characteristics of long-term, complexity and risk of military-civilian S&T collaborative innovation, this paper adopts the stochastic differential game model to study the strategy selection problem of military-civilian S&T collaborative innovation from a dynamic perspective.

The model takes a S&T collaborative innovation system consisting of the military (*M*) and the individual civilian enterprise (*C*) as the object of study. We consider the military enterprise to be affiliated with the military and do not consider the game relationship between the military enterprise and the military. We assume that the participants are perfectly rational and possess full information, and aim to maximize their own returns. Let *E*_*M*_(*t*) denote innovation effort level of the military at time *t*, and *E*_*C*_(*t*) denote the innovation effort level of the civilian enterprise at time *t*. The level of innovation effort of military and the civilian enterprise is positively correlated with their innovation costs. The higher the level of innovation effort, the higher the innovation costs.

Based on Gould [56], Huang et al. [57] and Lin et al. [58], the innovation costs are assumed to be:

$$\left\{ \begin{array}{l} C_{M}(t)=\frac{1}{2}\mu_{M}E_{M}^{2}(t)\\ C_{C}(t)=\frac{1}{2}\mu_{C}E_{C}^{2}(t) \end{array}\right.$$ (1)

where *μ*_*M*_ and *μ*_*C*_ respectively denote the innovation cost coefficients of the military and the civilian enterprise. *C*_*M*_(*t*) and *C*_*C*_(*t*) represent the cost of innovation for the military and the civilian enterprise, respectively.

In the process of S&T collaborative innovation, on the one hand, the participation strategies of civilian enterprises change in response to the market environment, R&D bottlenecks, and other internal and external risks, which in turn causes uncertainty in the level of S&T collaborative innovation. On the other hand, the complex procedures of military technology acquisition and the unfavorable situation of multiple steps and long cycle time of result transformation also indirectly affect the level of S&T collaborative innovation. It can be seen that uncertainties cause dynamic fluctuations in the level of S&T collaborative innovation. The deterministic game theory cannot fully reflect the real state of the subject’s decision-making behavior. In practical application, the stochastic process caused by random factors can generally be described approximately by the Brownian process, which obeys a normal distribution. Therefore, in this paper, we use the Brownian process to reflect the random disturbance factors in the process of the subject’s behavior and describe the stochastic change process of the level of scientific and technological collaborative innovation [54, 59]. We analyze the behavioral decisions of the military and the civilian enterprise to elucidate the changes in the stability of the system under stochastic disturbances. Referring to the related studies on differential game models [50, 60, 61], the stochastic differential equation was used to represent the change in the level of S&T collaborative innovation over time as:

$$\{\begin{array}{c} dG(t)=(\lambda_{M}E_{M}(t)+\lambda_{C}E_{C}(t)-\delta G(t))dt+\sigma G(t)dz(t)\\ G(0)=G_{0}\geq 0 \end{array}$$ (2)

where *G*(*t*) denotes the level of technological innovation at the time *t*. *G*(0) = *G*_0_≥0 represents the initial state of the system. *λ*_*M*_≥0 and *λ*_*C*_≥0 denote the coefficient of influence of the effort exerted by the military and the civilian enterprise on the level of technological innovation, respectively. *δ*>0 indicates the decay rate of the technology level in the technological innovation system, which is usually the relative decay rate of the technology being eliminated due to the rapid iteration of technological products and the incompatibility of the level of technological updates [48]. We set *σG*(*t*) as the random interference factors. *z*(*t*) is the standard Wiener process [50, 59], satisfying $\sigma (G(t))dz(t)=\sigma \sqrt{G}dz(t)$ [50, 62].

The total benefit level of the S&T collaborative innovation system *π*(*t*) at time *t* is expressed as:

$$\pi (t)=\alpha E_{M}(t)+\beta E_{C}(t)+\varepsilon G(t)$$ (3)

where *α*>0 and *β*>0 are the marginal revenue coefficients, which indicate the degree of impact of the efforts made by the military and the civilian enterprise on the overall revenue in S&T collaborative innovation, respectively. *ε*>0 is the S&T innovation impact factor representing the degree of impact of S&T collaborative innovation on the overall revenue.

In practice, from a self-interest perspective, enterprises are vulnerable to reduced effort or free-riding behavior. In order to promote continuous S&T collaborative innovation between the military and the civilian enterprise, to fully stimulate the willingness of the enterprise to cooperate in technological innovation, the military will subsidize to a certain extent the cost of technological innovation for the enterprise. We set the subsidy ratio as *θ*(*t*), whose value range is (0,1). The overall revenue obtained from the S&T collaborative innovation is distributed between the two according to the pre-negotiated distribution ratio, with the civilian enterprise receiving *ω* and the military receiving 1−*ω*, where *ω* is the allocation coefficient. The determination of the allocation coefficient should be decided according to the importance and contribution of the military and the civilian enterprise in the process of S&T collaborative innovation. In differential game problems, the discount rate can more accurately capture discounted returns in long-run planning [63]. The military and the civilian enterprise have the same discount rate *ρ*>0. Both parties have the goal of finding strategies for S&T innovation efforts that maximize their respective returns over an infinite time horizon.

The objective function for the military and the civilian enterprises can be expressed as [64]:

$$\max J_{M}=\int_{0}^{\infty }e^{-\rho t}[(1-\omega )(\alpha E_{M}(t)+\beta E_{C}(t)+\varepsilon G(t))-\frac{1}{2}\mu_{M}E_{M}^{2}(t)-\frac{1}{2}\theta (t)\mu_{C}E_{C}^{2}(t)]dt$$ (4)

$$\max J_{C}=\int_{0}^{\infty }e^{-\rho t}[\omega (\alpha E_{M}(t)+\beta E_{C}(t)+\varepsilon G(t))-\frac{1}{2}(1-\theta (t))\mu_{C}E_{C}^{2}(t)]dt$$ (5)

Where, the objective function of the military at time *t* consists of three parts: the first part is the benefit of participating in S&T collaborative innovation, the second part is the effort cost of participating in S&T collaborative innovation, and the third part is to share the cost of S&T innovation of civilian enterprises. The objective function of the civilian enterprise at time *t* consists of two parts: one part is the benefit of participating in S&T collaborative innovation, and the other part is the cost of participating in S&T collaborative innovation, including the cost of the enterprise’s own efforts and the cost of military subsidies.

There are three main control variables included in the differential game model: *E*_*M*_(*t*), *E*_*C*_(*t*) and *θ*(*t*). Since the model is difficult to solve under dynamically changing parameters, we assume that all model parameters are positive fixed constants, and are independent of time. In an infinite interval of time, the military and the civilian enterprise face the same game. Therefore, the equilibrium state corresponding to the static decisions of both participants is a static feedback equilibrium. To simplify the expression, the time variable *t* is omitted in the following study.

**Fig 1:**
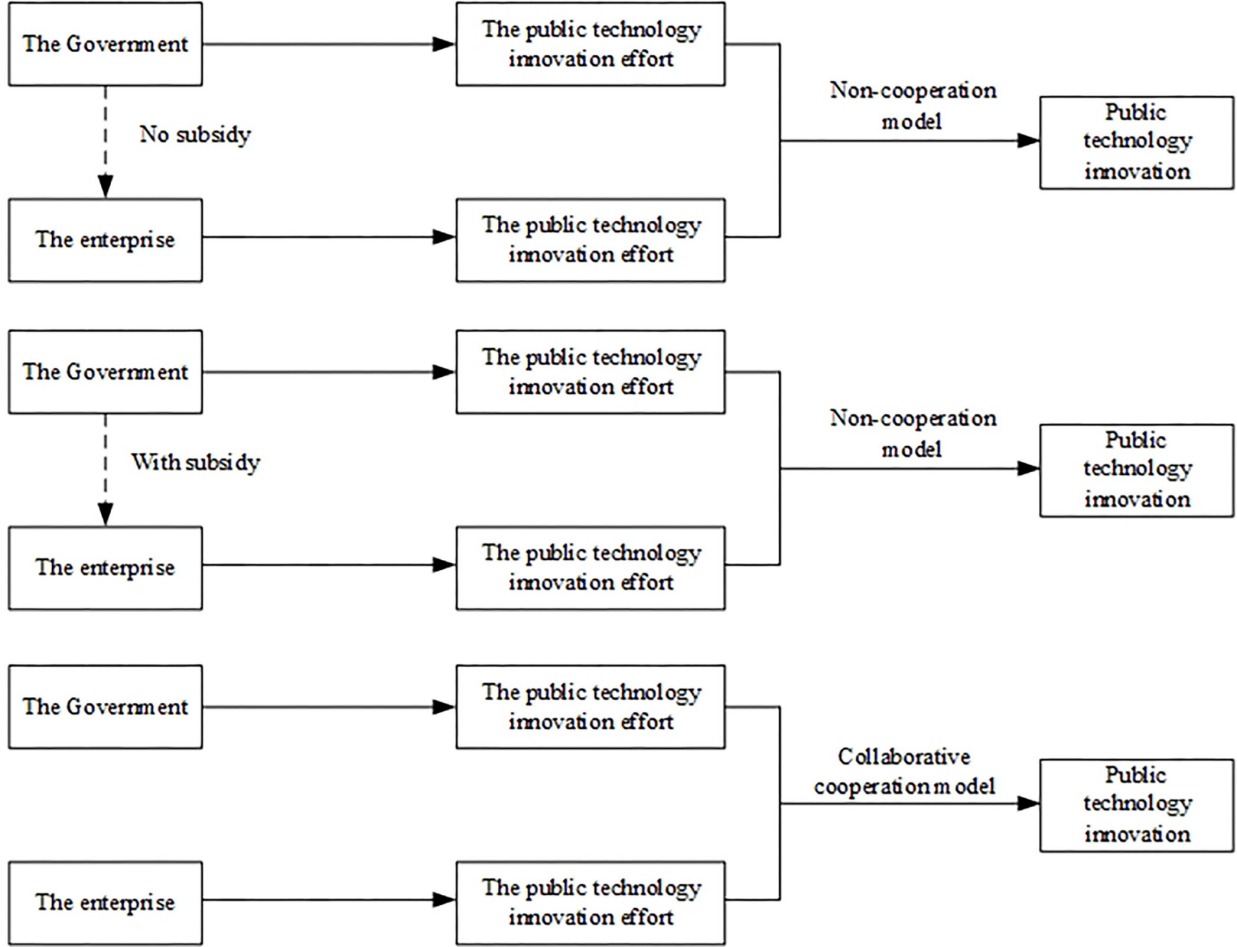


Combining with the actual situation, we divide the S&T collaborative innovation process in the context of integration into non-cooperation model without military subsidies, non-cooperation model with military subsidies and collaborative cooperation model, as shown in Fig 1.

### 3.1 Non-cooperative model without military subsidies (Model A)

In this case, the military and the civilian enterprise form an independent and equal competition relationship in the market. The military will not give subsidies to the civilian enterprise, and both sides will make independent and reasonable S&T innovation decisions with the goal of maximizing their own revenue. At this point the subsidy ratio *θ*(*t*) = 0, the objective functions of the military and the civilian enterprise are as follows:

$$\max J_{M}^{A}=\int_{0}^{\infty }e^{-\rho t}[(1-\omega )(\alpha E_{M}(t)+\beta E_{C}(t)+\varepsilon G(t))-\frac{1}{2}\mu_{M}E_{M}^{2}(t)]dt$$ (6)

$$\max J_{C}^{A}=\int_{0}^{\infty }e^{-\rho t}[\omega (\alpha E_{M}(t)+\beta E_{C}(t)+\varepsilon G(t))-\frac{1}{2}\mu_{C}E_{C}^{2}(t)]dt$$ (7)

To achieve the Nash equilibrium in this case, we assume that both the military and the civilian enterprise have optimal payoff functions *V*_*M*_(*G*) and *V*_*C*_(*G*), with continuously differentiable and bounded payoff functions. The *Hamilton-Jacobi-Bellman* equation exists for all *G*≥0 [63]:

$$\begin{array}{l} \rho V_{M}(G)=\underset{E_{M}\geq 0}{\max}[(1-\omega )(\alpha E_{M}+\beta E_{C}+\varepsilon G)-\frac{1}{2}\mu_{M}E_{M}^{2}\\ +V_{M}^{'}(G)(\lambda_{M}E_{M}+\lambda_{C}E_{C}-\delta G)+\frac{1}{2}\sigma^{2}V_{M}^{''}(G)] \end{array}$$ (8)

$$\begin{array}{l} \rho V_{C}(G)=\underset{E_{C}\geq 0}{\max}[\omega (\alpha E_{M}+\beta E_{C}+\varepsilon G)-\frac{1}{2}\mu_{C}E_{C}^{2}\\ +V_{C}^{'}(G)(\lambda_{M}E_{M}+\lambda_{C}E_{C}-\delta G)+\frac{1}{2}\sigma^{2}V_{C}^{''}(G)] \end{array}$$ (9)

To maximize profits, the optimal level of innovation effort for the military and the civilian enterprise is determined by *Bellman* theory of continuous dynamic programming [65]:

$$E_{M}^{A}=\frac{\left(1-\omega )\alpha +\lambda_{M}V_{M}^{'}(G\right)}{\mu_{M}},\;E_{C}^{A}=\frac{\omega \beta +\lambda_{C}V_{C}^{'}(G)}{\mu_{C}}$$ (10)

We take $E_{M}^{A}$ and $E_{C}^{A}$ into the HJB equation to obtain:

$$\begin{array}{l} \rho V_{M}(G)=[(1-\omega )\varepsilon -\delta V_{M}^{'}(G)]G+\frac{{\left[(1-\omega )\alpha +\lambda_{M}V_{M}^{'}(G)\right]}^{2}}{2\mu_{M}}\\ +\frac{\left[(1-\omega )\beta +\lambda_{C}V_{M}^{'}(G)][\omega \beta +\lambda_{C}V_{C}^{'}(G)\right]}{\mu_{C}} \end{array}$$ (11)

$$\begin{array}{l} \rho V_{C}(G)=(\varepsilon \omega -\delta V_{C}^{'}(G))G+{\frac{\left(\omega \beta +\lambda_{C}V_{C}^{'}(G)\right)}{2\mu_{C}}}^{2}\\ +\frac{\left[(1-\omega )\alpha +\lambda_{M}V_{M}^{'}(G)][\omega \alpha +V_{C}^{'}(G)\lambda_{M}\right]}{\mu_{M}} \end{array}$$ (12)

The above equation shows that the linear function with *G* as the independent variable is the solution of the HJB equation. Let *V*_*M*_(*G*) = *m*_1_*G*+*m*_2_ and *V*_*C*_(*G*) = *n*_1_*G*+*n*_2_ are satisfied for any *G*≥0, where *m*_1_, *m*_2_, *n*_1_, *n*_2_ are the constants to be solved. We plug the above expression into the HJB equation to solve for *m*_1_, *m*_2_, *n*_1_, *n*_2_:

$$\left\{ \begin{array}{l} m_{1}=\frac{(1-\omega )\varepsilon }{\rho +\delta }\\ m_{2}=\frac{{\left(1-\omega \right)}^{2}{\left[(\rho +\delta )\alpha +\lambda_{M}\varepsilon \right]}^{2}}{2\rho \mu_{M}{\left(\rho +\delta \right)}^{2}}+\frac{\left(1-\omega )\omega [(\rho +\delta )\beta +\lambda_{C}\varepsilon ][(\rho +\delta )\beta +\lambda_{C}\varepsilon \right]}{\rho \mu_{C}{\left(\rho +\delta \right)}^{2}}\\ n_{1}=\frac{\omega \varepsilon }{\rho +\delta }\\ n_{2}={\frac{\omega^{2}[(\rho +\delta )\beta +\lambda_{C}\varepsilon ]}{2\rho \mu_{C}{\left(\rho +\delta \right)}^{2}}}^{2}+\frac{\left(1-\omega )\omega [(\rho +\delta )\alpha +\lambda_{M}\varepsilon ][(\rho +\delta )\alpha +\lambda_{M}\varepsilon \right]}{\rho \mu_{M}{\left(\rho +\delta \right)}^{2}} \end{array}\right.$$ (13)

Combining the previous analysis, we can obtain the equilibrium results for the non-cooperative model without military subsidies. The optimal innovation effort levels of military and the civilian enterprise are:

$$E_{M}^{A*}=\frac{\left(1-\omega )[(\rho +\delta )\alpha +\lambda_{M}\varepsilon \right]}{(\rho +\delta )\mu_{M}},\;E_{C}^{A*}=\frac{\omega [(\rho +\delta )\beta +\lambda_{C}\varepsilon ]}{(\rho +\delta )\mu_{C}}$$ (14)

The optimal benefits for the military and the civilian enterprise are as follows.


$$V_{M}^{A*}(G)=\frac{(1-\omega )\varepsilon }{\rho +\delta }G+\frac{{\left(1-\omega \right)}^{2}{\left[(\rho +\delta )\alpha +\lambda_{M}\varepsilon \right]}^{2}}{2\rho \mu_{M}{\left(\rho +\delta \right)}^{2}}+\frac{(1-\omega )\omega {\left[(\rho +\delta )\beta +\lambda_{C}\varepsilon \right]}^{2}}{\rho \mu_{C}{\left(\rho +\delta \right)}^{2}}$$ (15)

$$V_{C}^{A*}(G)=\frac{\omega \varepsilon }{\rho +\delta }G+{\frac{\omega^{2}[(\rho +\delta )\beta +\lambda_{C}\varepsilon ]}{2\rho \mu_{C}{\left(\rho +\delta \right)}^{2}}}^{2}+\frac{(1-\omega )\omega {\left[(\rho +\delta )\alpha +\lambda_{M}\varepsilon \right]}^{2}}{\rho \mu_{M}{\left(\rho +\delta \right)}^{2}}$$ (16)

We further explore the dynamic development rules of S&T innovation. In Nash equilibrium, since the level of S&T innovation is influenced by random factors, we can obtain the expectation and variance of S&T innovation:

$$\sigma (G(t))dz(t)=\sigma \sqrt{G}dz(t)$$ (17)

$$\{\begin{array}{c} dG(t)=(\mu -\delta G(t))dt+\sigma G(t)dz(t)\\ G(0)=G_{0}\geq 0 \end{array}$$ (18)

According to the *Itô Lemma*, we can get:

$$\{\begin{array}{c} d{\left[G(t)\right]}^{2}=[(2\mu^{A}+\sigma^{2})G-2\delta G]dt+2G\sigma \sqrt{G}dz(t)\\ {\left[G(0)\right]}^{2}={\left(G_{0}\right)}^{2}\geq 0 \end{array}$$ (19)

*E*(*G*(*t*)) and *E*(*G*(*t*))^2^ satisfy the non-simultaneous linear differential equations:

$$\{\begin{array}{c} dE[G(t)]=[\mu^{A}-\delta E(G)]dt\\ E[G(0)]=G_{0} \end{array}$$ (20)

$$\{\begin{array}{c} dE{\left[G(t)\right]}^{2}=[(2\mu^{A}+\sigma^{2})G-2\delta E(G^{2})]dt\\ E{\left[G(0)\right]}^{2}={\left(G_{0}\right)}^{2} \end{array}$$ (21)

The expectation and variance can be expressed as follows.


$$\begin{array}{l} E^{A}[G(t)]=\frac{\mu^{A}}{\delta }+e^{-\delta t}(G_{0}-\frac{\mu^{A}}{\delta })\\ \underset{t\to \infty }{\lim}E^{A}[G(t)]=\frac{\mu^{A}}{\delta }\\ D^{A}[G(t)]=\frac{\sigma^{2}[\mu^{A}-2(\mu^{A}-\delta G_{0})e^{-\delta t}+(\mu^{A}-2\delta G_{0})e^{-2\delta t}]}{2\delta^{2}}\\ \underset{t\to \infty }{\lim}D^{A}[G(t)]=\frac{\sigma^{2}\mu^{A}}{2\delta^{2}}\\ \mu^{A}=\frac{\lambda_{M}(1-\omega )[(\rho +\delta )\alpha +\lambda_{M}\varepsilon ]}{(\rho +\delta )\mu_{M}}+\frac{\lambda_{C}\omega [(\rho +\delta )\beta +\lambda_{C}\varepsilon ]}{(\rho +\delta )\mu_{C}} \end{array}$$ (22)

### 3.2 Non-cooperative model with military subsidies (Model B)

In this case, the military will provide cost subsidies to promote the S&T innovation efforts of the civilian enterprise. First, the military decides its own S&T innovation effort level *E*_*M*_(*t*) and the cost subsidy ratio *θ*(*t*), and then the civilian enterprise decides its S&T innovation effort level *E*_*M*_(*t*) according to the cost subsidy ratio *θ*(*t*), and the objective of both parties’ decisions is to maximize their own benefits. The objective functions of the military and the civilian enterprise are as follows:

$$\max J_{M}^{B}=\int_{0}^{\infty }e^{-\rho t}[(1-\omega )(\alpha E_{M}(t)+\beta E_{C}(t)+\varepsilon G(t))-\frac{1}{2}\mu_{M}E_{M}^{2}(t)-\frac{1}{2}\theta (t)\mu_{C}E_{C}^{2}(t)]dt$$ (23)

$$\max J_{C}^{B}=\int_{0}^{\infty }e^{-\rho t}[\omega (\alpha E_{M}(t)+\beta E_{C}(t)+\varepsilon G(t))-\frac{1}{2}(1-\theta (t))\mu_{C}E_{C}^{2}(t)]dt$$ (24)

Using the inverse induction method, the objective function of the civilian enterprise is first solved, and then its optimal decision is substituted into the objective function of the military. The optimal revenue function *V*_*C*_(*G*) of the civilian enterprise satisfies the following *Hamilton-Jacobi-Bellman* equation.


$$\begin{array}{l} \rho V_{C}^{B}(G)=\underset{E_{C}\geq 0}{\max}[\omega (\alpha E_{M}+\beta E_{C}+\varepsilon G)-\frac{1}{2}(1-\theta )\mu_{C}E_{C}^{2}\\ +V_{C}^{'}(G)(\lambda_{M}E_{M}+\lambda_{C}E_{C}-\delta G)+\frac{1}{2}\sigma^{2}V_{C}^{''}(G)] \end{array}$$ (25)

Similarly, the *Bellman* theory of continuous dynamic programming has been used to determine the optimal level of innovation effort for the civilian enterprise.


$$E_{C}^{B}=\frac{\omega \beta +\lambda_{C}V_{C}^{'}(G)}{(1-\theta )\mu_{C}}$$ (26)

The optimal payoff function $V_{M}^{B}(G)$ for the military satisfies the following Hamilton-Jacobi-Bellman equation.


$$\begin{array}{l} \rho V_{M}^{B}(G)=\underset{E_{M}\geq 0}{\max}[(1-\omega )(\alpha E_{M}+\beta E_{C}+\varepsilon G)-\frac{1}{2}\mu_{M}E_{M}^{2}(t)-\frac{1}{2}\theta (t)\mu_{C}E_{C}^{2}(t)\\ +V_{M}^{'}(G)(\lambda_{M}E_{M}+\lambda_{C}E_{C}-\delta G)+\frac{1}{2}\sigma^{2}V_{M}^{''}(G)] \end{array}$$ (27)

We bring $E_{C}^{B}$ into $\rho V_{M}^{B}(G)$ to get the military’s optimal level of innovation effort $E_{M}^{B}$ and subsidy rate *θ*^*B*^ as follows.


$$\begin{array}{l} E_{M}^{B}=\frac{\left(1-\omega )\alpha +\lambda_{M}V_{M}^{'}(G\right)}{\mu_{M}}\\ \theta^{B}=\frac{\left(2-3\omega )\beta +\lambda_{C}(2V_{M}^{'}(G)-V_{C}^{'}(G)\right)}{\left(2-\omega )\beta +\lambda_{C}(2V_{M}^{'}(G)+V_{C}^{'}(G)\right)} \end{array}$$ (28)

We bring $E_{M}^{B}$, $E_{C}^{B}$ and *θ*^*B*^ to *ρV*_*C*_(*G*) and *ρV*_*M*_(*G*) to obtain the following result.


$$\begin{array}{l} \rho V_{M}(G)={\frac{\left[(1-\omega )\alpha +\lambda_{M}V_{M}^{'}(G)\right]}{2\mu_{M}}}^{2}+\frac{{\left[(2-\omega )\beta +\lambda_{C}(2V_{M}^{'}(G)+V_{C}^{'}(G))\right]}^{2}}{8\mu_{C}}\\ +[(1-\omega )\varepsilon -\delta V_{M}^{'}(G)]G \end{array}$$ (29)

$$\begin{array}{l} \rho V_{C}(G)=\frac{\left[(1-\omega )\alpha +\lambda_{M}V_{M}^{'}(G)][\omega \alpha +\lambda_{M}V_{C}^{'}(G)\right]}{\mu_{M}}\\ +\frac{\left[\omega \beta +\lambda_{C}V_{C}^{'}(G)][(2-\omega )\beta +\lambda_{C}(2V_{M}^{'}(G)+V_{C}^{'}(G))\right]}{4\mu_{C}}+(\varepsilon \omega -\delta V_{C}^{'}(G))G \end{array}$$ (30)

Similarly, the above equation shows that the linear function with *G* as the independent variable is the solution of the HJB equation. Let *V*_*M*_(*G*) = *m*_1_*G*+*m*_2_ and *V*_*C*_(*G*) = *n*_1_*G*+*n*_2_ are satisfied for any *G*≥0. We take the above expression into the HJB equation to solve for *m*_1_, *m*_2_, *n*_1_, *n*_2_.


$$\left\{ \begin{array}{l} m_{1}=\frac{(1-\omega )\varepsilon }{\rho +\delta }\\ m_{2}=\frac{{\left(1-\omega \right)}^{2}{\left[(\rho +\delta )\alpha +\lambda_{M}\varepsilon \right]}^{2}}{2\rho {\left(\rho +\delta \right)}^{2}\mu_{M}}+\frac{{\left(2-\omega \right)}^{2}{\left[\beta (\rho +\delta )+\lambda_{C}\varepsilon \right]}^{2}}{8\rho {\left(\rho +\delta \right)}^{2}\mu_{C}}\\ n_{1}=\frac{\omega \varepsilon }{\rho +\delta }\\ n_{2}=\frac{(1-\omega )\omega {\left[\alpha (\rho +\delta )+\lambda_{M}\varepsilon \right]}^{2}}{\rho {\left(\rho +\delta \right)}^{2}\mu_{M}}+\frac{(2-\omega )\omega {\left[(\rho +\delta )\beta +\lambda_{C}\varepsilon \right]}^{2}}{4\rho {\left(\rho +\delta \right)}^{2}\mu_{C}} \end{array}\right.$$ (31)

The optimal innovation effort levels of military and the civilian enterprise are:

$$\begin{array}{l} E_{M}^{B*}=\frac{\left(1-\omega )[(\rho +\delta )\alpha +\lambda_{M}\varepsilon \right]}{(\rho +\delta )\mu_{M}},E_{C}^{B*}=\frac{\left(2-\omega )[(\rho +\delta )\beta +\lambda_{C}\varepsilon \right]}{2(\rho +\delta )\mu_{C}}\\ \theta^{B}=\left\{ \begin{array}{l} \frac{2-3\omega }{2-\omega }(0<\omega <\frac{2}{3})\\ 0(\frac{2}{3}\leq \omega <1) \end{array}\right. \end{array}$$ (32)

The optimal benefits for the military and the civilian enterprise are as follows.


$$\begin{array}{l} V_{M}^{B*}(G)=\frac{(1-\omega )\varepsilon }{\rho +\delta }G+\frac{{\left(1-\omega \right)}^{2}{\left[(\rho +\delta )\alpha +\lambda_{M}\varepsilon \right]}^{2}}{2\rho {\left(\rho +\delta \right)}^{2}\mu_{M}}+\frac{{\left(2-\omega \right)}^{2}{\left[\beta (\rho +\delta )+\lambda_{C}\varepsilon \right]}^{2}}{8\rho {\left(\rho +\delta \right)}^{2}\mu_{C}}\\ V_{C}^{B*}(G)=\frac{\omega \varepsilon }{\rho +\delta }G+\frac{(1-\omega )\omega {\left[\alpha (\rho +\delta )+\lambda_{M}\varepsilon \right]}^{2}}{\rho {\left(\rho +\delta \right)}^{2}\mu_{M}}+\frac{(2-\omega )\omega {\left[(\rho +\delta )\beta +\lambda_{C}\varepsilon \right]}^{2}}{4\rho {\left(\rho +\delta \right)}^{2}\mu_{C}} \end{array}$$ (33)

The expectation and variance can be expressed as follows.


$$\begin{array}{l} E^{B}[G(t)]=\frac{\mu^{B}}{\delta }+e^{-\delta t}(G_{0}-\frac{\mu^{B}}{\delta })\\ \underset{t\to \infty }{\lim}E^{B}[G(t)]=\frac{\mu^{B}}{\delta }\\ D^{B}[G(t)]=\frac{\sigma^{2}[\mu^{B}-2(\mu^{B}-\delta G_{0})e^{-\delta t}+(\mu^{B}-2\delta G_{0})e^{-2\delta t}]}{2\delta^{2}}\\ \underset{t\to \infty }{\lim}D^{B}[G(t)]=\frac{\sigma^{2}\mu^{B}}{2\delta^{2}}\\ \mu^{B}=\frac{\lambda_{M}(1-\omega )[(\rho +\delta )\alpha +\lambda_{M}\varepsilon ]}{(\rho +\delta )\mu_{M}}+\frac{\lambda_{C}(2-\omega )[(\rho +\delta )\beta +\lambda_{C}\varepsilon ]}{2(\rho +\delta )\mu_{C}} \end{array}$$ (34)

### 3.3 Collaboration model (Model C)

In this case, the military and the civilian enterprise carry out S&T collaborative innovation and jointly decide the level of S&T innovation efforts. The decision goal of both parties is to maximize the system benefits. At this moment, the cost subsidy of the military to civilian enterprises can be regarded as the internal fund transfer of the collaborative innovation system. The system objective function is as follows.


$$\max J^{C}=\int_{0}^{\infty }e^{-\rho t}[(\alpha E_{M}(t)+\beta E_{C}(t)+\varepsilon G(t))-\frac{1}{2}\mu_{M}E_{M}^{2}(t)-\frac{1}{2}\mu_{C}E_{C}^{2}(t)]dt$$ (35)

The optimal benefit function of the system *V*(*G*) satisfies the following *Hamilton-Jacobi-Bellman* equation.


$$\begin{array}{l} \rho V(G)=\underset{E_{M}\geq 0,E_{C}\geq 0}{\max}[(\alpha E_{M}(t)+\beta E_{C}(t)+\varepsilon G(t))-\frac{1}{2}\mu_{M}E_{M}^{2}(t)-\frac{1}{2}\mu_{C}E_{C}^{2}(t)\\ +V^{'}(G)(\lambda_{M}E_{M}+\lambda_{C}E_{C}-\delta G)+\frac{1}{2}\sigma^{2}V^{''}(G)] \end{array}$$ (36)

Similarly, the *Bellman* theory of continuous dynamic programming has been used to determine the optimal level of innovation effort for the civilian enterprise.


$$E_{M}^{C}=\frac{\alpha +\lambda_{M}V^{'}(G)}{\mu_{M}},\;E_{C}^{C}=\frac{\beta +\lambda_{C}V^{'}(G)}{\mu_{C}}$$ (37)

We bring $E_{M}^{C}$ and $E_{C}^{C}$ into the HJB equation to obtain the result as follows.


$$\rho V(G)=\underset{E_{M}\geq 0,E_{C}\geq 0}{\max}[{\frac{\left(\alpha +\lambda_{M}V^{'}(G)\right)}{2\mu_{M}}}^{2}+{\frac{\left(\beta +\lambda_{C}V^{'}(G)\right)}{2\mu_{C}}}^{2}+(\varepsilon -\delta V^{'}(G))G+\frac{1}{2}\sigma^{2}V^{''}(G)]$$ (38)

The above equation shows that the linear function with *G* as the independent variable is the solution of the HJB equation. Let *V*(*G*) = *p*_1_*G*+*p*_2_ is satisfied for any *G*≥0.We bring the above expression into the HJB equation to solve for *p*_1_, *p*_2_.


$$\left\{ \begin{array}{l} p_{1}=\frac{\varepsilon }{\rho +\delta }\\ p_{2}={\frac{\left[\alpha (\rho +\delta )+\lambda_{M}\varepsilon \right]}{2\rho (\rho +\delta )\mu_{M}}}^{2}+{\frac{\left[\beta (\rho +\delta )+\lambda_{C}\varepsilon \right]}{2\rho (\rho +\delta )\mu_{C}}}^{2} \end{array}\right.$$ (39)

The optimal innovation effort levels of military and the civilian enterprise are:

$$E_{M}^{C*}=\frac{\alpha (\rho +\delta )+\lambda_{M}\varepsilon }{\mu_{M}(\rho +\delta )},\;E_{C}^{C*}=\frac{\beta (\rho +\delta )+\lambda_{C}\varepsilon }{\mu_{C}(\rho +\delta )}$$ (40)

The optimal benefits for the military and the civilian enterprise are as follows.


$$\begin{array}{l} V_{M}^{C*}(G)=(1-\omega )[\frac{\varepsilon }{\rho +\delta }G+{\frac{\left[\alpha (\rho +\delta )+\lambda_{M}\varepsilon \right]}{2\rho (\rho +\delta )\mu_{M}}}^{2}+{\frac{\left[\beta (\rho +\delta )+\lambda_{C}\varepsilon \right]}{2\rho (\rho +\delta )\mu_{C}}}^{2}]\\ V_{C}^{C*}(G)=\omega [\frac{\varepsilon }{\rho +\delta }G+{\frac{\left[\alpha (\rho +\delta )+\lambda_{M}\varepsilon \right]}{2\rho (\rho +\delta )\mu_{M}}}^{2}+{\frac{\left[\beta (\rho +\delta )+\lambda_{C}\varepsilon \right]}{2\rho (\rho +\delta )\mu_{C}}}^{2}] \end{array}$$ (41)

The expectation and variance can be expressed as follows.


$$\begin{array}{l} E^{C}[G(t)]=\frac{\mu^{C}}{\delta }+e^{-\delta t}(G_{0}-\frac{\mu^{C}}{\delta })\\ \underset{t\to \infty }{\lim}E^{C}[G(t)]=\frac{\mu^{C}}{\delta }\\ D^{C}[G(t)]=\frac{\sigma^{2}[\mu^{C}-2(\mu^{C}-\delta G_{0})e^{-\delta t}+(\mu^{C}-2\delta G_{0})e^{-2\delta t}]}{2\delta^{2}}\\ \underset{t\to \infty }{\lim}D^{C}[G(t)]=\frac{\sigma^{2}\mu^{C}}{2\delta^{2}}\\ \mu^{C}=\frac{\lambda_{M}[\alpha (\rho +\delta )+\lambda_{M}\varepsilon ]}{\mu_{M}(\rho +\delta )}+\frac{\lambda_{C}[\beta (\rho +\delta )+\lambda_{C}\varepsilon ]}{\mu_{C}(\rho +\delta )} \end{array}$$ (42)

## 4 Equilibrium analysis

Combining the above model projection results, we compare the equilibrium strategies, optimal benefits and steady states of the S&T innovation system of the military and the civilian enterprise under three different models, and explore the conditions for S&T innovation cooperation between the military and the civilian enterprise, as well as the impact of the intensity of random disturbances on the decisions of both parties.

### 4.1 Equilibrium analysis of optimal innovation effort level

The optimal level of innovation effort of military and the civilian enterprise is positively correlated with the marginal revenue coefficient. Thus, the higher the level of innovation effort exerted by the military and the civilian enterprise, the greater the impact of S&T innovation. We take Model A as an example for further analysis. When S&T innovation projects are less difficult and complex, the discounting rate *ρ* and the technology degradation rate *δ* tend to be higher. At this time there may be $\frac{(1-\omega )\alpha }{\mu_{M}}\gg \frac{(1-\omega )\lambda_{M}\varepsilon }{(\rho +\delta )\mu_{M}}$, $\frac{\omega \beta }{(\rho +\delta )\mu_{C}}\gg \frac{\omega \lambda_{C}\varepsilon }{(\rho +\delta )\mu_{C}}$. In this case, the marginal revenue coefficients of both parties have a weaker influence on the decision about the optimal level of innovation effort. When S&T innovation projects are more difficult and complex, the discounting rate *ρ* and the technology degradation rate *δ* are typically lower. At this time there may be $\frac{(1-\omega )\alpha }{\mu_{M}}\ll \frac{(1-\omega )\lambda_{M}\varepsilon }{(\rho +\delta )\mu_{M}}$, $\frac{\omega \beta }{(\rho +\delta )\mu_{C}}\ll \frac{\omega \lambda_{C}\varepsilon }{(\rho +\delta )\mu_{C}}$. In this case, the marginal benefit coefficients of both parties play a crucial role in determining the optimal level of innovation effort. Although the military does not provide subsidies to the civilian enterprise, it can also motivate them to increase the level of innovation effort.

Firstly, we compare the optimal innovation effort levels of the military and the civilian enterprise in the three models to obtain $E_{M}^{A*}=E_{M}^{B*}<E_{M}^{C*}$. It can be found that the military has not reduced its level of innovation effort by paying a cost subsidy. When the subsidy ratio *θ*^*B*^ is positive $\left(0<\omega <\frac{2}{3}\right)$, we have $E_{C}^{A*}<E_{C}^{B*}<E_{C}^{C*}$. Military cost subsidies can increase the level of innovation effort of the civilian enterprise. Thus, military subsidies are effective in providing innovation incentives to the civilian enterprise. However, the military’s cost subsidy rate is tied to the revenue allocation ratio. Therefore, the military will only subsidize the costs of the civilian enterprise if *ω* in the range of $\left[0,\frac{2}{3}\right]$.

Comparing models A and B, cost subsidies can effectively guide the civilian enterprise to invest in S&T innovation efforts, which further leads to an improved level of S&T collaborative innovation and mitigates the double marginal effects under the no-subsidy decision. The respective and total S&T innovation levels of military and the civilian enterprise achieve partial Pareto improvements under the military subsidy. Further analysis shows that the optimal effort of the civil enterprise in the non-cooperative mode is related to its revenue allocation ratio, innovation impact ratio, marginal revenue ratio and cost ratio, whereas in the collaborative mode, the optimal effort of the enterprise is not related to the revenue allocation ratio. It can be observed that in the process of collaborative innovation, the optimal effort of the enterprise is no longer influenced by the revenue allocation ratio, but depends more on its own capability.

### 4.2 Equilibrium analysis of optimal benefits

First, a comparative analysis of the overall optimal level of benefits under different models shows that when $0<\omega <\frac{2}{3}$, $V_{M+C}^{A*}<V_{M+C}^{B*}<V_{M+C}^{C*}$. The contrast shows that the total optimal revenue level is highest in the S&T collaborative innovation model, next in the model with military subsidies, and lowest in the model without military subsidies. Accordingly, S&T collaborative innovation between the military and civilian enterprises is conducive to improving the overall optimal revenue level of the system.

Then, we compare and analyze the optimal level of benefits for the military under different models to obtain that when $0<\omega <\frac{2}{3}$, $V_{M}^{A*}<V_{M}^{B*}$ must hold. That is, compared to the model without military subsidies, the optimal level of benefits for the military is higher in the model with military subsidies.

At last, we compare and analyze the optimal level of benefits for the civilian enterprise under different models to obtain that when $0<\omega <\frac{2}{3}$, $V_{C}^{A*}<V_{C}^{B*}$ must hold. That is, the optimal level of returns for civilian firms is higher in the model with military subsidies than in the model without military subsidies. However, further comparison requires a disaggregated discussion of the revenue allocation factors.

Let $\varphi_{M}=\frac{{\left[(\rho +\delta )\alpha +\lambda_{M}\varepsilon \right]}^{2}}{\rho \mu_{M}{\left(\rho +\delta \right)}^{2}}$, $\varphi_{C}={\frac{\left[(\rho +\delta )\beta +\lambda_{C}\varepsilon \right]}{2\rho \mu_{C}{\left(\rho +\delta \right)}^{2}}}^{2}$. Owing to the highest overall optimal benefit level in the case of S&T collaborative innovation, the difference between the benefit levels of the military and the civilian enterprise in different modes is mainly related to the benefit allocation coefficient and *φ*_*M*_、*φ*_*C*_. By designing a reasonable benefit distribution scheme, the optimal benefit distribution between military and civilian enterprises can be achieved, so that both sides can realize their optimal technology sharing benefits in the collaborative innovation mode are higher than other modes. At this point, collaborative innovation is Pareto optimal for both sides. Thus, to coordinate the technology sharing behavior of both sides, the benefit allocation coefficients need to be discussed.

From $V_{M}^{C*}-V_{M}^{B*}>0$ and $V_{C}^{C*}-V_{C}^{B*}>0$, we have $\frac{2\varphi_{M}}{4\varphi_{M}+\varphi_{C}}\leq \omega \leq \frac{4\varphi_{M}}{4\varphi_{M}+\varphi_{C}}$. Due to $0<\omega <\frac{2}{3}$, we get $0<\frac{2\varphi_{M}}{4\varphi_{M}+\varphi_{C}}<\frac{1}{2}<\frac{2}{3}$. Therefore, when $\frac{4\varphi_{M}}{4\varphi_{M}+\varphi_{C}}\geq \frac{2}{3}$, the range of values of the benefit distribution coefficient is $\left[\frac{2\varphi_{M}}{4\varphi_{M}+\varphi_{C}},\frac{2}{3}\right)$. When $\frac{4\varphi_{M}}{4\varphi_{M}+\varphi_{C}}<\frac{2}{3}$, the range of values of the benefit distribution coefficient is $\left[\frac{2\varphi_{M}}{4\varphi_{M}+\varphi_{C}},\frac{4\varphi_{M}}{4\varphi_{M}+\varphi_{C}}\right]$. In the reasonable range of the benefit distribution coefficients, there are $V_{C}^{A*}<V_{C}^{B*}<V_{C}^{C*}$ and $V_{M}^{A*}<V_{M}^{B*}<V_{M}^{C*}$ hold. At this point the system is able to achieve optimal Pareto for both parties involved in S&T collaborative innovation.

Although the total benefits of both military and the civilian enterprise under the collaborative cooperation model are higher than the total benefits under the two non-cooperative models of no-cost subsidy and cost subsidy, they will not choose to cooperate if the benefit allocation within the system is unreasonable and the benefit distribution is unclear, so a reasonable benefit allocation coefficient must be determined to achieve individual Pareto optimality for the military and civilian enterprises.

### 4.3 Analysis of system stability

We further analyze the trend of expectation and variance over continuous time. When $\delta >\frac{\mu^{A}}{G_{0}}$, $\frac{\partial E^{A}[G(t)]}{\partial t}<0$. The level of technological innovation effort declines over time, and both the military and the civilian enterprise require a continuous increase their effort to ensure effectiveness. When $\delta <\frac{\mu^{A}}{G_{0}}$, $\frac{\partial E^{A}[G(t)]}{\partial t}>0$. The level of S&T innovation effort increases over time. Thus, the military and the civilian enterprise can maintain a stable level of S&T innovation effort without additional effort. When $\delta >\frac{\mu^{A}}{G_{0}}$ and $t>\frac{\ln2}{\delta }$, $\frac{\partial D^{A}[G(t)]}{\partial t}<0$. The variance of the level of S&T collaborative innovation gradually decreases over time, and the effect of random disturbances on the stability of the system is slight. When $\delta <\frac{\mu^{A}}{G_{0}}$ or $\delta >\frac{\mu^{A}}{G_{0}}$ and $t<\frac{\ln2}{\delta }$, $\frac{\partial D^{A}[G(t)]}{\partial t}>0$. The variance of the level of S&T innovation gradually increases with time. At present, random disturbances have a greater impact on the level of S&T innovation, and the military and the civilian enterprise should further improve their ability to cope with the risk.

The results of comparing the expected value and variance of the level of S&T collaborative innovation and the stability value under the three models are:

$$E^{C}[G(t)]>E^{B}[G(t)]>E^{A}[G(t)],\;\underset{t\to \infty }{\lim}E^{C}[G(t)]>\underset{t\to \infty }{\lim}E^{B}[G(t)]>\underset{t\to \infty }{\lim}E^{A}[G(t)]$$

$$D^{C}[G(t)]>D^{B}[G(t)]>D^{A}[G(t)],\;\underset{t\to \infty }{\lim}D^{C}[G(t)]>\underset{t\to \infty }{\lim}D^{B}[G(t)]>\underset{t\to \infty }{\lim}D^{A}[G(t)]$$

Compared with the unsubsidized model, the level of S&T innovation under the military-subsidized model is improved, but the variance increases. In comparison with the first two models, the S&T innovation level under the collaborative innovation model has the dynamic evolution characteristics of maximum expectation and variance, which is due to the fact that the military does not perform its management and regulation functions under the collaborative model of military and civilian enterprises, resulting in greater risk and uncertainty faced by the system. Although the maximum expected level of innovation is achieved in the collaborative innovation model, in practice, the military is generally risk neutral, so for risk-averse civilian enterprises can choose collaborative innovation, while risk-averse civilian enterprises will tend to choose the non-cooperative model. If the military wants to reach a higher level of S&T innovation through the collaborative innovation model, it must strengthen the management of the stability and continuity of the system to motivate civilian enterprises to participate in the collaboration.

## 5 Numerical analysis

In order to verify the reasonableness of the above conclusions, we conducted numerical simulation experiment. In the process of military-civilian S&T collaborative innovation, it is assumed that the military’s technological innovation efforts can bring about greater technological innovation and benefit enhancement, as the military is more dominant. The relationship *λ*_*M*_>*λ*_*C*_, *α*>*β* and *μ*_*M*_>*μ*_*C*_ hold. Under the premise of the basic assumptions of the model, drawing on the research results in the school-enterprise cooperation and innovation system [46], combined with the actual military-civilian S&T collaborative innovation, the parameters are set as follows: *λ*_*M*_ = 0.6, *λ*_*C*_ = 0.4, *α* = 0.7, *β* = 0.4, *μ*_*M*_ = 0.6, *μ*_*C*_ = 0.3, *G*_0_ = 10, *t* = 80, *ρ* = 0.1, *δ* = 0.1, *ε* = 0.1, *σ* = 0.1. We disaggregate the S&T innovation levels by referring to the relevant literature [59]: $G(t+\Delta t)=G(t)+\Delta t(\mu -\delta G(t))+\sigma \sqrt{G(t)}\sqrt{\Delta t}Z(t)$, where *Z*(*t*)~*N*(0,1) is a standard normally distributed variable.

### 5.1 Stochastic evolutionary simulation analysis of S&T innovation level

Given the stochastic disturbance factor *σ* = 0.1, the above relevant parameters were substituted into the decision expression for the level of technological innovation, and the evolution characteristics of the supply of epidemic prevention materials and their expectation and variance under the three cases were analyzed by using MATLAB software.

**Fig 2:**
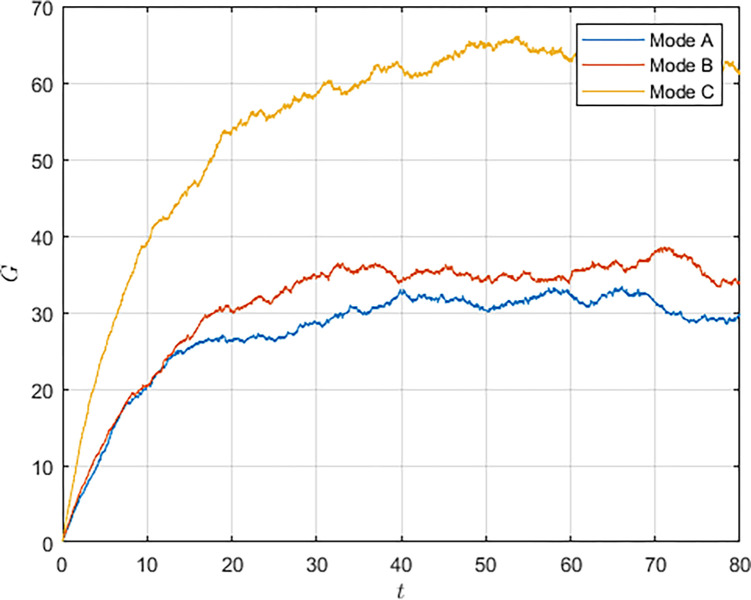


First, we simulated the evolution of the S&T innovation level over time, as shown in Fig 2. Due to the long-term and complex characteristics of S&T innovation and the interference of random factors in the innovation process, the evolution of the S&T innovation level shows fluctuations. However, the innovation level still shows an incremental trend over time and eventually reaches a stable state. Comparing the three models, it can be seen that in the cooperation model, the S&T innovation level grows the fastest and reaches the highest S&T innovation level, whereas in the non-cooperation model, military subsidies can increase the growth rate of the S&T innovation level and make the system reach the S&T innovation level in a shorter period of time.

**Fig 3:**
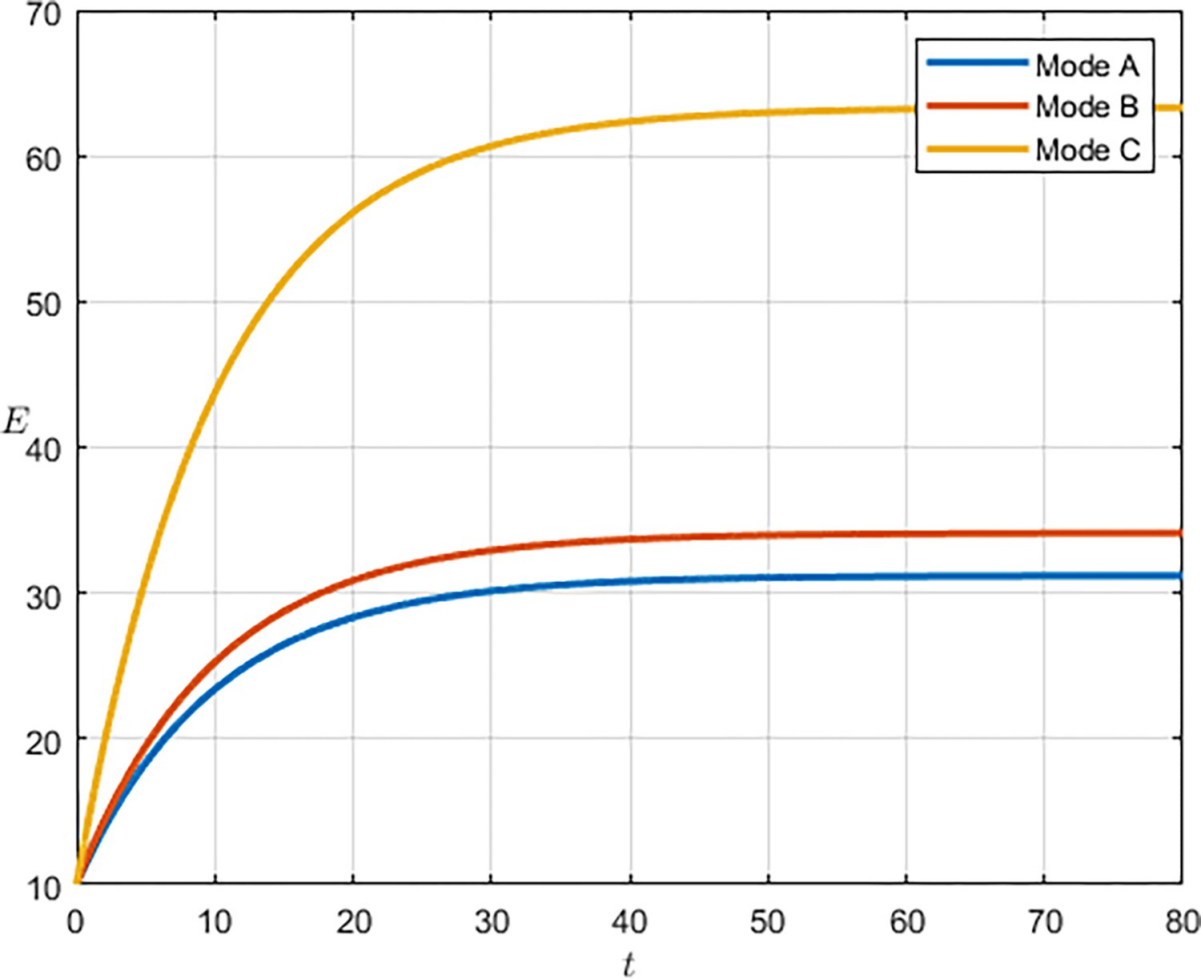


**Fig 4:**
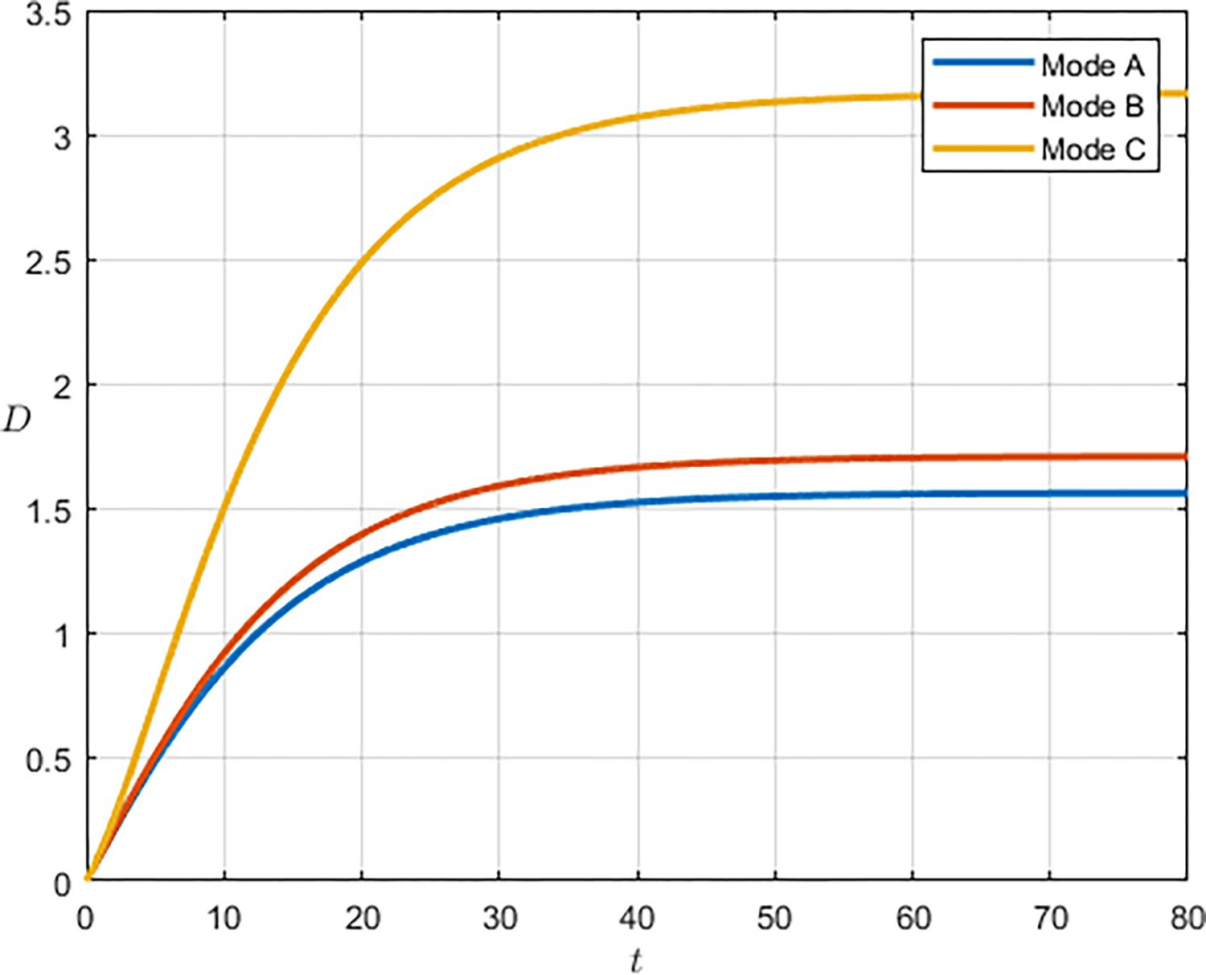


As shown in Figs 3 and 4, we have simulated the trend of S&T innovation level expectation and variance evolution over time. The expectation of S&T innovation level grows slowly under the no-subsidy model, whereas under the collaborative cooperation model, it is able to achieve a rapid growth of S&T innovation level, which eventually reaches approximately twice as much as the expectation under the non-cooperation model. Although in reality, the S&T innovation level is subject to random disturbing factors and deviates from the expected value, it can maintain a floating range with a confidence level of about 95%. Therefore, the military and civilian enterprises can make decisions on the S&T innovation model according to the range in which the expected value is located.

Compared with the non-cooperative model, S&T innovation under the cooperative model is exposed to more risks and random disturbance factors, and its stability is worse, which indicates that the development of S&T innovation is significantly influenced by the innovation model. Under the cooperative model, both the military and the civilian enterprise take the overall benefit of the system as the decision target, which lowers the threshold of technology transfer and improves the efficiency and level of S&T innovation. However, the lack of supervision and management functions of the military in the collaborative cooperation model makes S&T innovation less stable.

### 5.2 Comparative analysis of the benefits of S&T innovation

**Fig 5:**
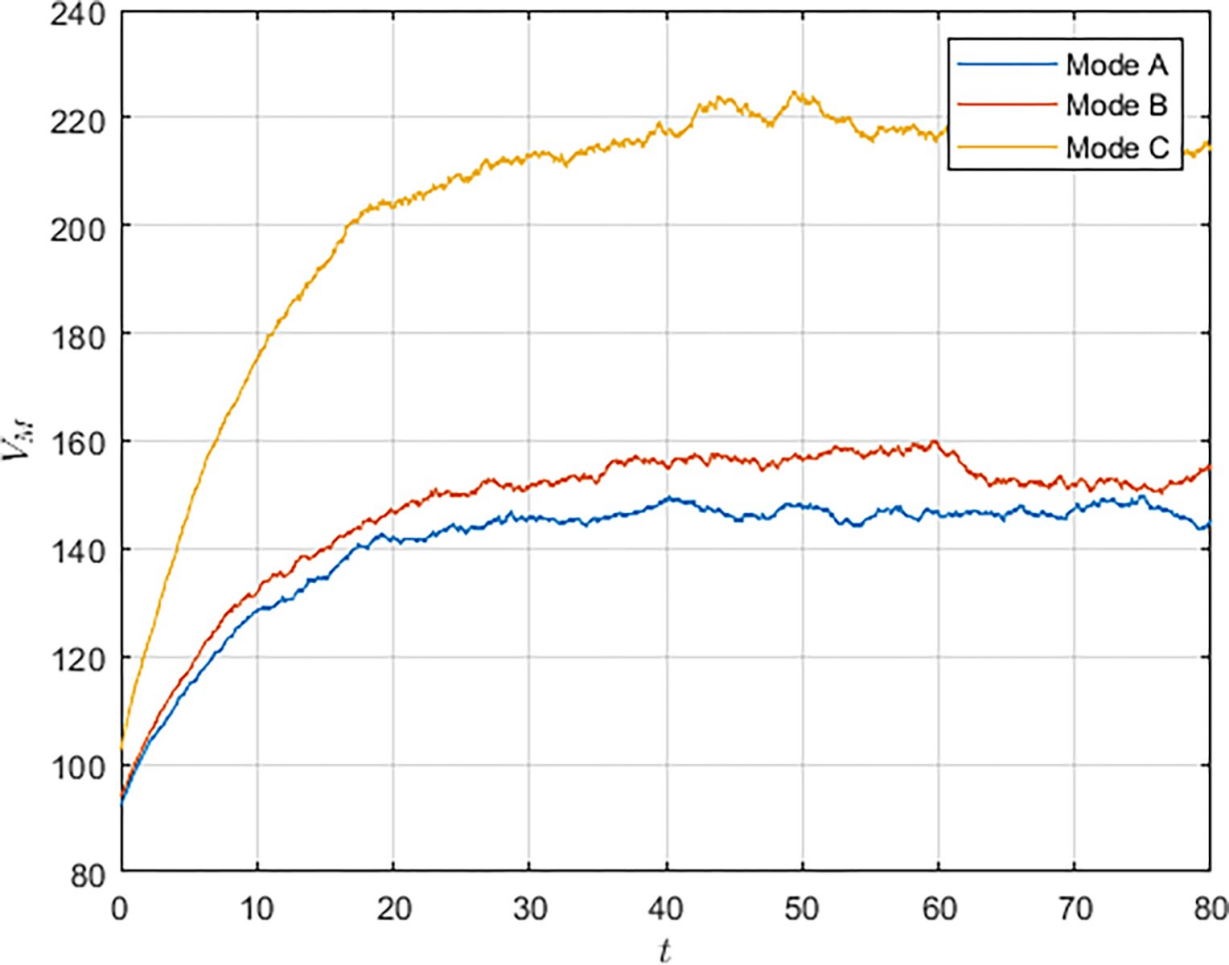


**Fig 6:**
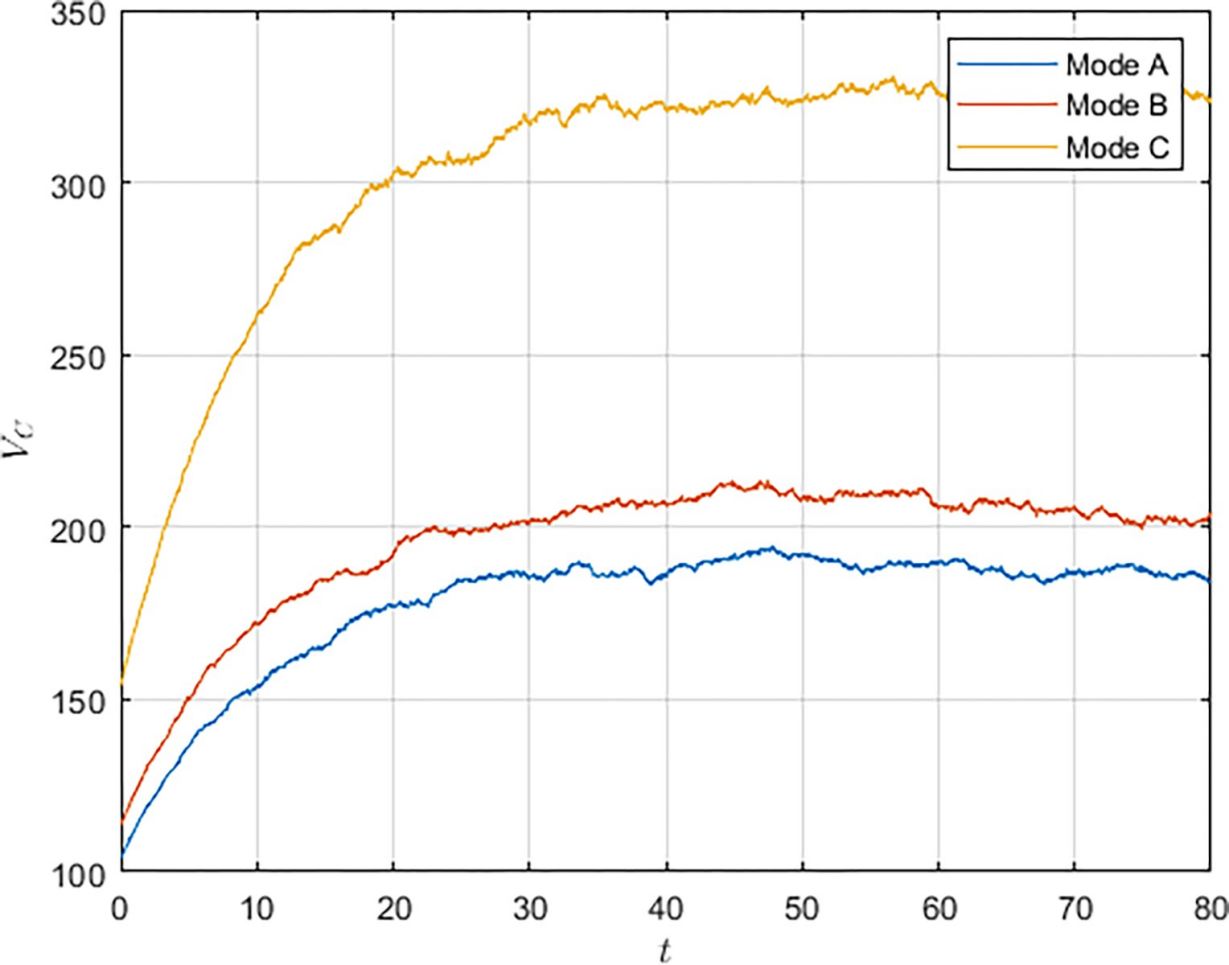


We set the revenue allocation coefficient *ω* = 0.6, which is able to satisfy the Pareto optimality condition. Figs 5 and 6 simulate the evolution of benefits for the military and the civilian enterprise under different models, respectively. For both military and the civilian enterprise, the benefits under the collaborative model are much higher than those under the non-cooperative model.

**Fig 7:**
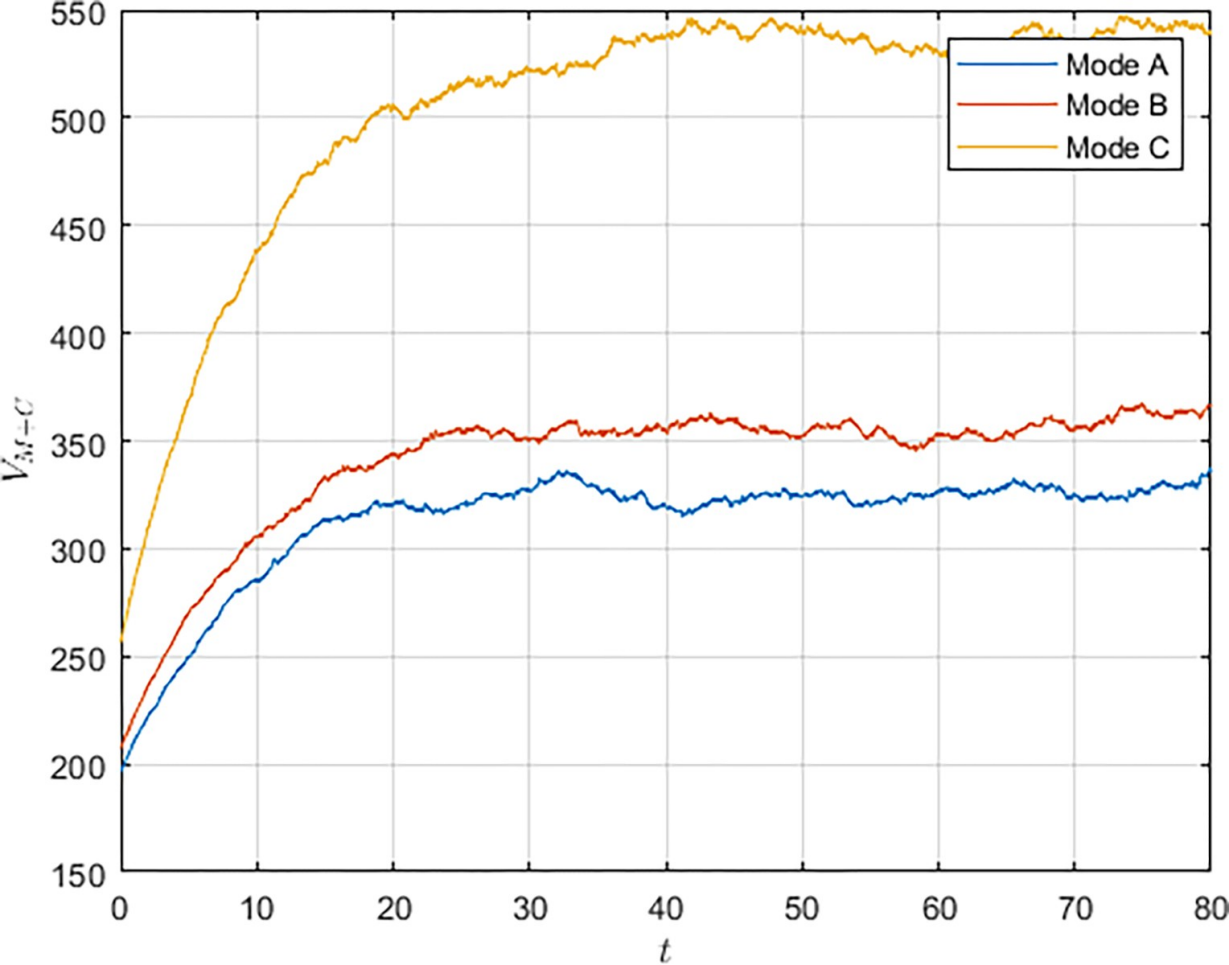


As shown in Fig 7, when the military and the civilian enterprise collaborate in innovation, the overall system benefits are much larger than the overall benefits in the non-cooperation mode, and they can achieve the fastest growth of system benefits and reach a stable state. For the military, actively guiding civilian enterprises to participate in S&T collaborative innovation and formulating a reasonable benefit distribution scheme can achieve a stable situation of mutual benefit and win-win for both sides.

### 5.3 Analysis of the effectiveness of military-subsidized Pareto improvements

**Fig 8:**
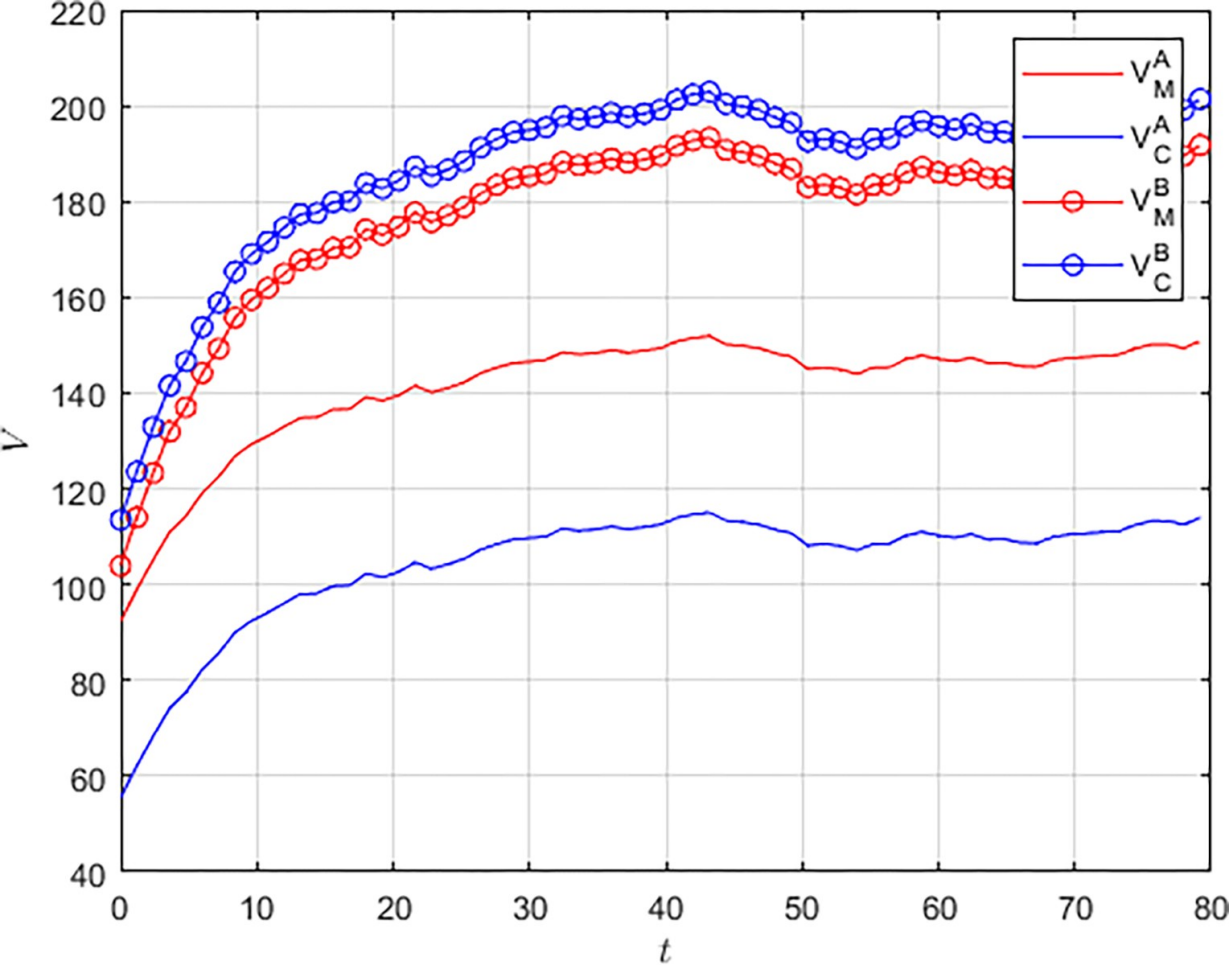


To further explore the importance of cost subsidies in S&T innovation, we separately compare the Pareto improvement effects of military subsidies. Numerical simulation is used to compare the level of benefits for military and civilian enterprises in the no-military-subsidy model and the military-subsidy model over the same time horizon. As shown in Fig 8, military cost subsidies can significantly improve the revenue level of both the military and the civilian enterprise, and the degree of revenue improvement is greater for the civilian enterprise. The military can share the innovation risk with the civilian enterprise and mitigate the double marginal effect of the no-subsidy model, so that both the military and the civilian enterprise can achieve Pareto improvements.

### 5.4 Analysis of the effects of random disturbance variables

**Fig 9:**
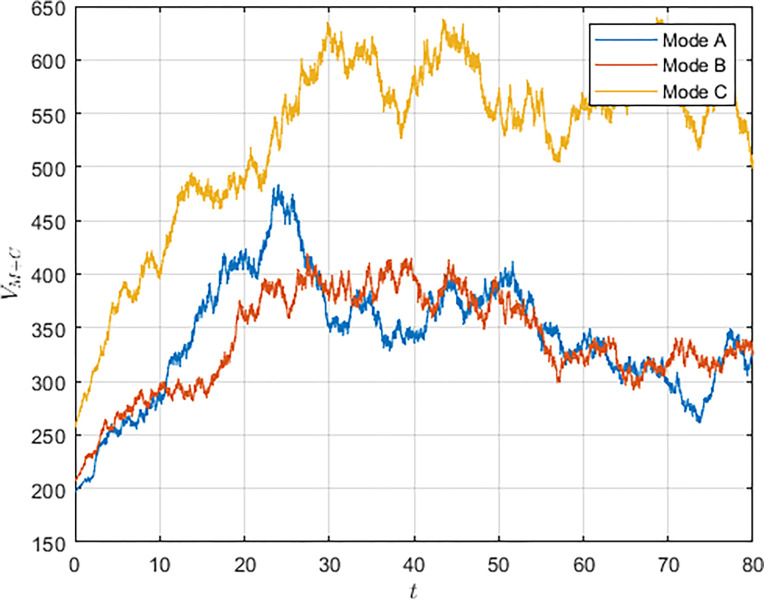


**Fig 10:**
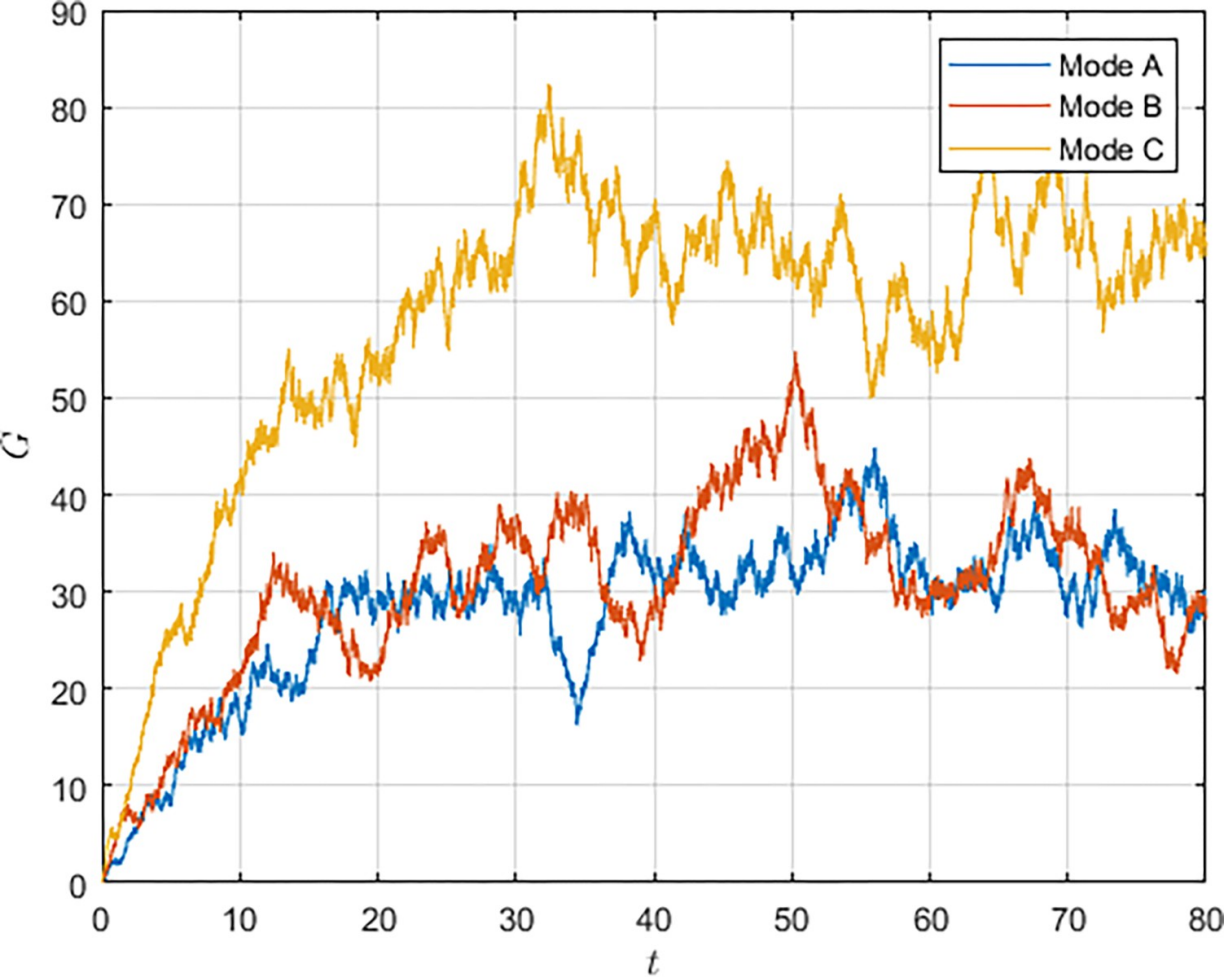


To further explore the effects of random disturbance variables on S&T innovation levels and returns, we analyzed the evolution of S&T innovation levels and overall system returns over time when the random disturbance variable *σ* = 0.6. From Figs 9 and 10, it can be obtained that under the influence of disturbance factors, the STI level and overall system revenue maintain the overall trend of growth and eventually reach a steady state. As the intensity of the disturbance increases, the Pareto improvement effect of the military subsidy receives a shock. At discrete time points, the innovation level or the overall revenue level in model B may be lower than that in model A. The decisions of the military and the civilian enterprise may change with the influence of external uncertainties, leading to the destabilization of the system. Therefore, the military needs to strengthen the response capability of the S&T innovation system to resist external uncertainty risk factors.

## 6 Conclusions and policy recommendations

S&T innovation, as the key to achieving the goal of an integrated national strategic system and capability, is particularly important in military competition. As an effective way to improve S&T innovation capability, military-civilian collaborative S&T innovation is inevitably affected by uncertainties such as technology iteration, R&D risks, transfer barriers, and enterprise risk preferences. Different from previous studies, this paper, with the help of stochastic differential game theory, incorporates the dynamic development of military-civilian collaborative innovation into a unified research framework. The results of the study indicate that:

1. The level of S&T innovation and overall revenue is lower in the non-cooperative mode without military subsidies than in the cooperative mode.
2. In the non-cooperative model with military subsidies, cost subsidies, as an effective adjustment mechanism, can lead to an increase in the benefits of both the military-civilian S&T collaborative innovation system and the participating parties. The military can adjust the subsidies to civilian enterprises according to the strategic needs of national defense and market changes.
3. S&T collaborative innovation is conducive to improving the level of S&T innovation and the optimal revenue of the system. Within a reasonable range of values of the revenue allocation coefficient, the system can achieve the Pareto optimum for both parties to participate in S&T collaborative innovation. The collaborative cooperation mode is the best strategy for military-civilian S&T collaborative innovation. The range of values of the benefit allocation coefficients should be reasonably set to achieve the Pareto optimum of the system and the participating subjects.
4. The optimal effort of civil enterprises in the non-cooperation mode is related to the revenue allocation coefficient, but in the collaborative cooperation mode, the optimal effort of civil enterprises is not related to the revenue allocation coefficient, which may reflect that in the process of scientific and technological collaborative innovation, the optimal effort of enterprises is no longer influenced by the revenue allocation coefficient, but depends more on their own ability.
5. Compared with the no subsidy mode, the level of S&T innovation under the military subsidy mode is improved, but the variance increases. Risk-averse civilian firms may choose cooperative innovation, while risk-averse civilian firms will tend to choose the non-cooperative mode.

In light of the above conclusions, we propose the following policy recommendations:

1. Adopt appropriate subsidy policies to guide enterprises to participate in military-civilian S&T collaborative innovation. For civilian enterprises, the military subsidy policy can alleviate the pressure of innovation cost to a certain extent, disperse the impact of double marginal effect, and prompt enterprises to increase the investment in S&T innovation driven by interests. In addition to cost subsidies, loan subsidies and capital injection can also be used in various ways to provide the necessary guarantee conditions for civilian enterprises to complete the collaborative innovation of military-civilian S&T.
2. Strengthen the system construction of military-civilian S&T collaborative innovation and stimulate the innovation vitality of civilian enterprises. The riskiness of innovation greatly hinders civilian enterprises to participate in military-civilian S&T collaborative innovation. Strengthening the system construction of information sharing, price mechanism to promote effective, risk sharing and win-win benefit is conducive to alleviating the innovation pressure of civilian enterprises and forming the traction effect of S&T collaborative innovation. In particular, it is necessary to sort out and rationalize the interests between the subjects of military-civilian S&T collaborative innovation, improve the benefit distribution mechanism, objectively and accurately evaluate the value of the cooperation results, and distribute the total revenue according to the degree of contribution of the subjects, so as to protect the reasonable interests of both military and civilian parties.
3. Improve the convergence of supporting policies and optimize the internal and external environment of collaborative innovation. By formulating and improving measures for the transformation of military-civilian S&T achievements and intellectual property protection policies, and further strengthening policy guidance for civilian high-tech enterprises, we can enhance the strength of military-civilian S&T collaborative innovation and reduce the internal and external risks of the collaborative innovation system.

The study in this paper still has some limitations:

1. This paper assumes that all parameters in the model are constant and independent of time, which can be followed for the non-degenerate problem of differential equation.
2. In this paper, military enterprises are regarded as affiliated organizations of the military, and the game relationship between them is not considered. The subsequent study can be extended to the problem of military-led, military-civilian S&T collaborative innovation with the participation of military enterprises and civilian enterprises.
3. In reality, the military-civilian S&T collaborative innovation system is often more complex and involves the participation of multiple civilian enterprises. The competition and cooperation relationship of multiple participants deserves further research.
